# Vaccination rate and symptoms of long COVID among dental teams in Germany

**DOI:** 10.1038/s41598-025-96670-8

**Published:** 2025-04-21

**Authors:** Maria Mksoud, Till Ittermann, Birte Holtfreter, Andreas Söhnel, Carmen Söhnel, Alexander Welk, Sebastian Paris, Florentina Sophie Melzow, Annette Wiegand, Philipp Kanzow, Andrea Rau, Stefan Kindler, Thomas Kocher

**Affiliations:** 1 Department of Oral and Maxillofacial Surgery/Plastic Surgery, University Medicine Greifswald, Walther-Rathenau-Str. 42a, 17475 Greifswald, Germany; 2 Institute for Community Medicine, University Medicine Greifswald, Greifswald, Germany; 3 Department of Restorative Dentistry, Periodontology and Endodontology, University Medicine Greifswald, Greifswald, Germany; 4 Department of Prosthodontics, Gerodontology and Biomaterials, University Medicine Greifswald, Greifswald, Germany; 5 Department of Operative, Preventive and Pediatric Dentistry, University Medicine Berlin, Charité, Berlin, Germany; 6 Department of Preventive Dentistry, Periodontology and Cariology, University Medical Center Göttingen, Göttingen, Germany

**Keywords:** SARS-CoV-2, Long COVID, Occupational risk, Dentistry, Public health, Occupational health, Dental epidemiology

## Abstract

Although COVID-19 is no longer a global public health threat, its consequences persist, with long COVID affecting at least 10% of patients and manifesting in various organ systems. National and international health agencies promoted vaccination to enhance population immunity, prioritizing healthcare personnel due to their high occupational risk. In a previous study, we found that the risk of SARS-CoV-2 transmission among dental teams in Germany was not higher than in the general population. This follow-up investigation aims to assess the vaccination status and the prevalence and severity of long COVID symptoms among dental teams in Germany. As part of a follow-up investigation involving the original cohort, 267 team members from 186 German dental practices previously included in the initial study completed an online questionnaire. The questionnaire covered three topics: (1) vaccination status, (2) confirmed COVID-19 diagnosis, and (3) self-reported long COVID symptoms. One hundred and seventy-two dentists (64.4%), 74 dental assistants (27.7%) and 21 dental hygienists (7.9%) completed the questionnaire. In total, 245 participants (91.8%) were at least once vaccinated. A COVID-19 infection after January 1st 2021 was reported by 146 (54.7%) participants, of which 33 participants (22.6%) suffered from long COVID symptoms. Our results showed lower vaccination rates among dental auxiliary personnel compared to dentists (95.9% vs. 84.2%). Individuals with long COVID symptoms were more often dental assistants (48.5% vs. 29.2%) or dental hygienists (15.2% vs. 8.0%) than dentists (36.4% vs. 62.8%) compared to the group not reporting long COVID symptoms (*p* = 0.025). In addition, it is unlikely that dental healthcare personnel are more prone to experiencing more severe symptoms compared to the general population. Vaccination against SARS-CoV-2 is likely to help against symptoms of long COVID.

**Supplementary Information:**

The online version contains supplementary material available at 10.1038/s41598-025-96670-8.

## Introduction

The severe acute respiratory syndrome coronavirus 2 (SARS-CoV-2) has infected approximately 775 million people globally (as of August 5th 2024)^1^. Initial efforts to contain the spread of the virus focused primarily on preventive measures such as physical distancing and mandatory face masks^2^. On December 21st 2020, the European Commission conditionally authorized the first vaccine against the coronavirus disease (COVID-19) for people aged 16 years and older^3^. Subsequently, several vaccines have been authorized and administered worldwide^4^. Public awareness campaigns around the world emphasized the importance of immediate vaccination. Those campaigns were based on numerous studies reporting various benefits of the COVID-19 vaccine including enhanced protection from severe complications, reduced need for hospitalization and mechanical ventilation^5,6^. As of November 2023, approximately 67% of the world’s population had received a complete primary series of a COVID-19 vaccine^7^. This marked the transition from the pandemic response phase to pandemic control.

Despite the decline of corona-related hospital admissions and deaths, the consequences of the pandemic still persist^8,9^. These consequences manifest as multisystem conditions with diverse symptoms following an infection with the SARS-CoV-2 virus, often referred to as post-acute sequelae of COVID-19 or long COVID^10^. Long COVID is defined by the World Health Organization (WHO) as the continuation or development of new symptoms at least three months after COVID-19 infection and lasting for at least two months with no sufficient explanation by alternative diagnoses^11^. It is roughly estimated that at least 10% of individuals infected by SARS-CoV-2 suffer from long COVID, although the real number is probably much higher due to undocumented and untreated cases^12,13^. Fatigue, exhaustion, dyspnea, and lack of concentration are constantly reported as the most common long COVID symptoms^14^. Due to the lack of effective treatment approaches, this condition poses a significant challenge to healthcare systems in both the short and long-term. Furthermore, the additional costs associated with protective measures, such as screening, testing, and personal protective equipment, have placed a substantial economic burden on healthcare facilities, and private practitioners alike^15,16^. A complicating factor when analyzing the prevalence of long COVID is accurately defining the onset time, whether before or after COVID infection. Some of those symptoms may have been mildly present before COVID-19 infection but worsened afterwards.

Dental personnel are known to work in the oral cavity, a body region with potentially high respiratory viral and bacterial load. Due to the high number of dental procedures resulting in the spread of infectious respiratory particles, dental healthcare personnel were thought to suffer from an increased risk of exposure to SARS-CoV-2^17^. At the early stages of the pandemic, healthcare authorities worldwide issued various guidelines concerning the virus and its transmission routes. These guidelines, based on little research and information, varied significantly, presenting challenges for patients and healthcare providers. Since then, occupational risk among dental healthcare personnel has been well examined^18^, confirming that with appropriate infection control measures, the risk of infection can be mitigated^19^. Nevertheless, the particularity of the setting in the dental praxis with the fear of contracting the virus from proximate patients and infectious respiratory particles generated during dental procedures increased the psychological pressure on oral healthcare personnel since the outbreak of the pandemic^20^. Psychological distress has been reported to contribute to physical complaints particularly among dental healthcare personnel at the beginning of the pandemic^21,22^.

In a previous study conducted between January and March 2021, we examined the hazard of SARS-CoV-2 infection among German dental teams (*n* = 2784 team members of 186 German dental practices). The overall test positivity rate for SARS-CoV-2 antibodies among dental healthcare workers was 5.2%. We have followed up this population to assess the coronavirus vaccination rate and symptoms of long COVID based on an online questionnaire.

## Materials and methods

### Recruitment

In a previous study, we analyzed data from 2784 individuals who worked at 1390 licensed dental practices in 5 urban regions in Germany between January and April 2021^23^. During the study period, we obtained their consent to contact them again for a follow-up investigation. The follow-up study protocol was approved by the Ethical Committee of the University Medicine Greifswald (BB 081/20b October 06th 2022). All participants signed an informed consent. All study procedures were conducted in accordance with the ethical standards as laid down in the Declaration of Helsinki.

Between November and December 2022, we re-invited the participants from the baseline study to participate in a follow-up investigation. The invitation included a link to answer an online questionnaire with the same numerical identifier (ID) used during the previous study to ensure data privacy and enable the matching of information regarding previous infection and vaccination from both investigations. Our follow-up sample consisted of 267 participants (Supplementary Fig. 1).

## Data collection/online questionnaire

The online questionnaire was divided into three parts. The first part focused on the vaccination status of the participants including the number of doses received and the type of vaccine used. The second part consisted of information on confirmed COVID-19 diagnosis based on polymerase chain reaction (PCR) assays and the reason for taking the test, as well as the course of infection and need of hospitalization or admission to intensive care unit (ICU). The third part evaluated long COVID symptoms reported by the participants and the impact of these symptoms on their daily life. We inquired information about prevalent symptoms known to be associated with coronavirus disease including fatigue, exhaustion, dyspnea (shortness of breath), coughing, lack of concentration, headache, dysgeusia (distortion of the sense of taste), anosmia (partial or full loss of smell), muscle pain, limb pain, chest pain, joint pain, sleeping disorder, depression heart stumble, and palpitation^24^. The severity of the symptoms was categorized based on the impact on daily activities as follows: (1) No impairment, (2) Negligible impairment, (3) Moderate impairment, (4) Severe impairment, (5) care needed. Reported long COVID symptoms as well as severity of symptoms were based on subjective perception only. All procedures complied with national privacy and data protection laws in Germany and were reviewed in advance by the University Medicine Greifswald’s data protection officer.

### Statistical analysis

Continuous data was reported by means and standard deviations (SD). Categorical data was described by absolute numbers and percentages. Differences in continuous variables across groups were evaluated by t-tests, while differences in categorical variables were tested by Chi squared tests. A *p* < 0.05 was considered as statistically significant. Data was analyzed with Stata 18.0.

## Results

**Fig. 1:**
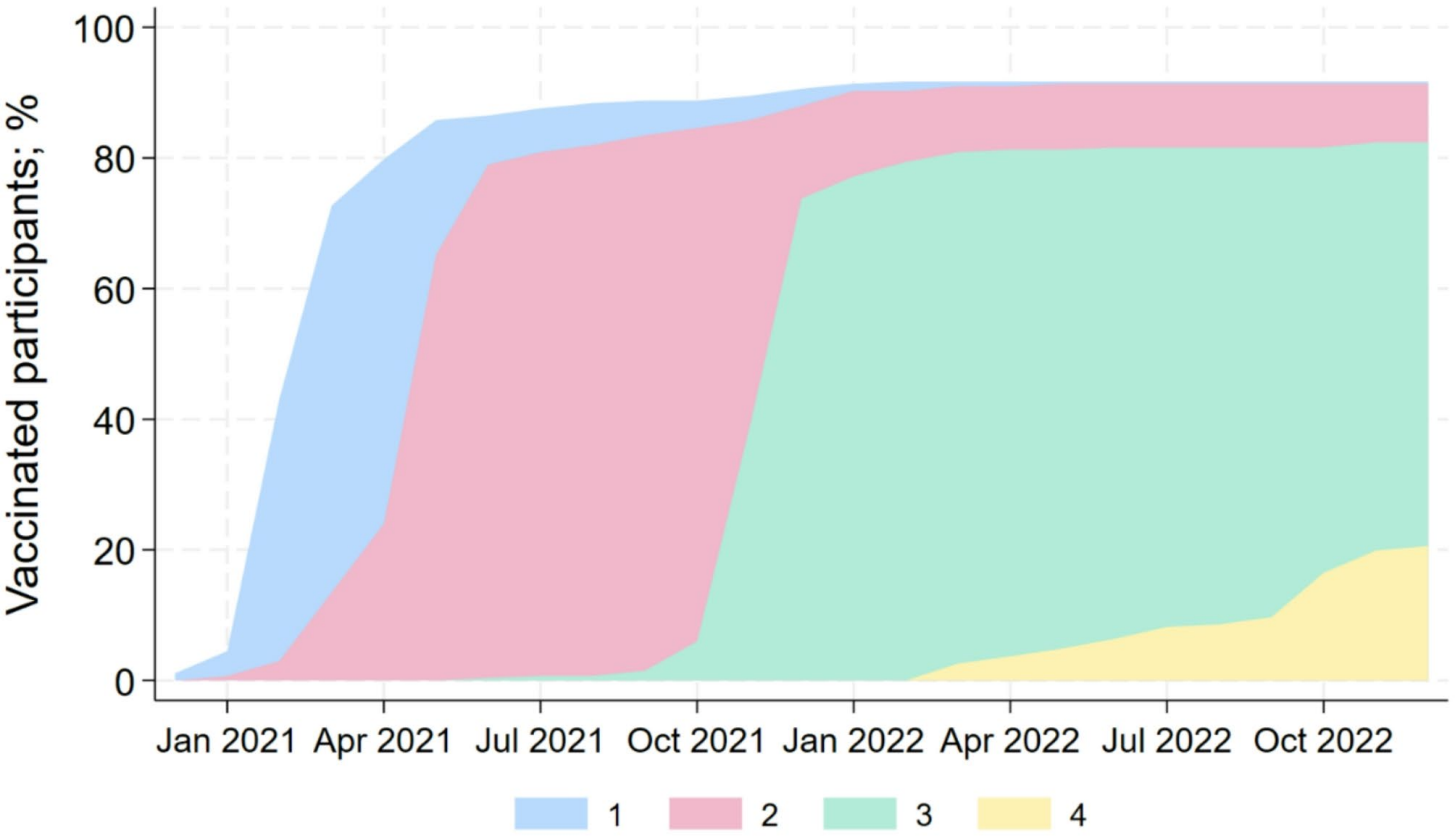
Percentage of vaccinated participants according to the number of vaccination doses (coloring) during the study period (*n* = 267).

We received responses from 201 women and 66 men aged 18 to 73 years (mean age 49 years; SD 10 years) from 186 dental practices. Among the participants were 172 dentists (64.4%), 74 dental assistants (27.7%), and 21 dental hygienists (7.9%); Table 1. Overall, 245 (91.8%) participants were at least once vaccinated, 244 (91.4%) twice vaccinated, 219 (82.0%) three times vaccinated, and 54 (20.2%) four times vaccinated (Fig. 1). The vaccination frequency differed significantly between dentists (95.9%) and dental auxiliary personnel (i.e. dental assistants and hygienists; 84.2%) (*p* = 0.001).

**Table 1:** Characteristics of all participants reporting a confirmed COVID-19 diagnosis (*n* = 146) and of the subgroup reporting to suffer from at least one long COVID symptom symptom (*n* = 33).

	Total	Long COVID		*P* value*
		Yes	No	
N	146 (100%)	33 (22.6%)	113 (77.4%)	
Sex				
Female	113 (82.5%)	27 (87.1%)	86 (81.1%)	0.442
Male	24 (17.5%)	4 (12.9%)	20 (18.9%)	
Vaccination status				
Vaccinated	129 (88.4%)	27 (81.8%)	102 (90.3%)	0.183
Unvaccinated	17 (11.6%)	6 (18.2%)	11 (9.7%)	
Occupational group				
Dentist	83 (56.8%)	12 (36.4%)	71 (62.8%)	0.025
Dental assistants	49 (33.6%)	16 (48.5%)	33 (29.2%)	
Dental hygienists	14 (9.6%)	5 (15.2%)	9 (8.0%)	

Data are expressed as absolute numbers and percentages. *Chi squared tests.

Since January 1st 2021, 146 (54.7%) participants reported COVID-19 infection as ascertained by a PCR testing. Infections were more common among dental auxiliary personnel (66.3%) than dentists (48.3%; *p* = 0.005). Of the 146 participants infected with COVID, 33 (22.0%) participants reported suffering from at least one long COVID symptom. The mean age was not significantly different between those with (46.3 years; SD 10.3) and those without (47.5 years; SD 11.1) long COVID symptoms (*p* = 0.588), nor was the percentage of women. The frequency of women was slightly higher but not significantly different in the long COVID group compared to individuals not reporting long COVID symptoms (87.1% vs. 81.1%; *p* = 0.442). Individuals with long COVID symptoms were more often dental assistants (long COVID 48.5% vs. no long COVID 29.2%) or dental hygienists (15.2% vs. 8.0%) than dentists (36.4% vs. 62.8%) compared to the group not reporting long COVID symptoms (*p* = 0.025). The frequency of COVID-19-vaccination was slightly lower in individuals with long COVID symptoms than in individuals without long COVID symptoms (81.8% vs. 90.3%; *p* = 0.183).

**Fig. 2:**
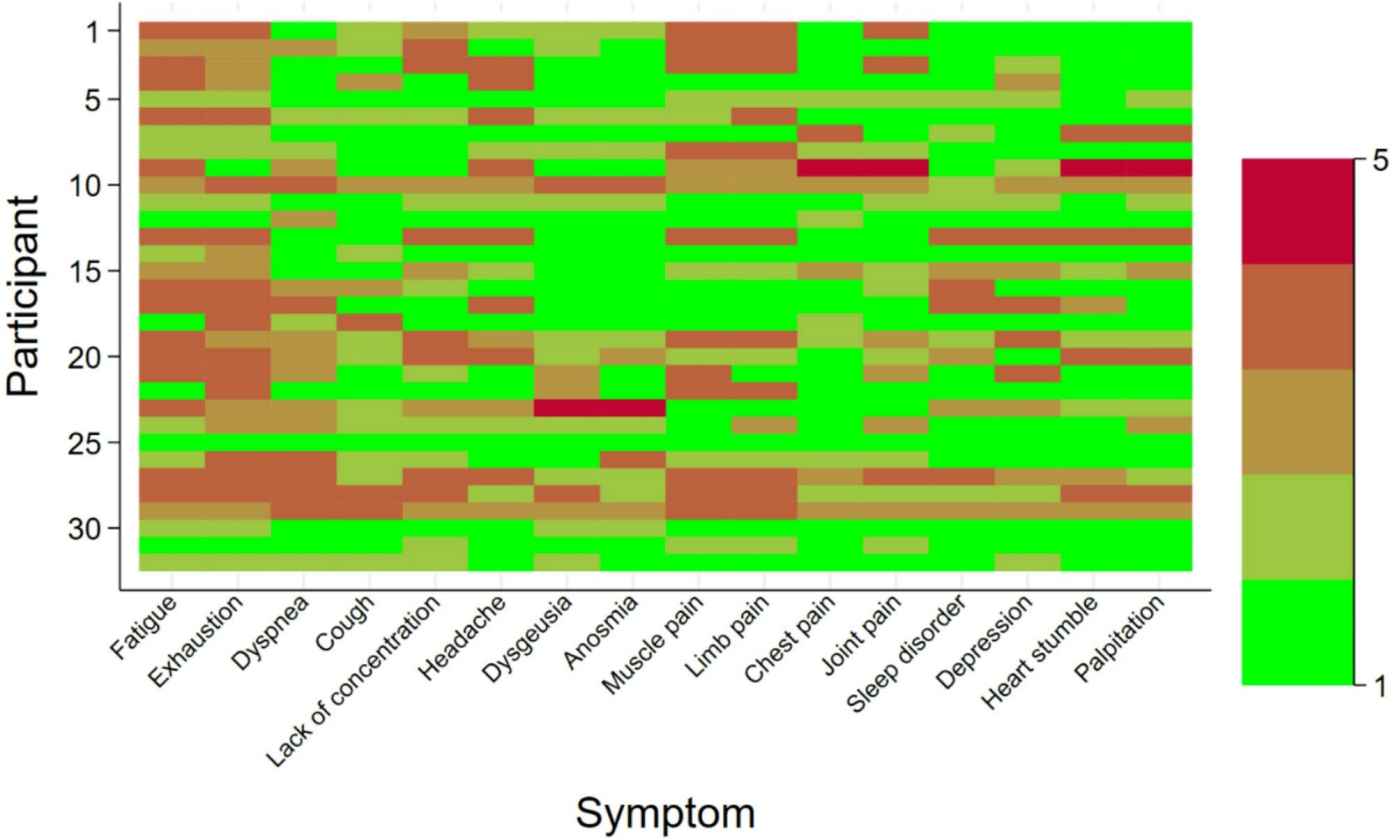
Frequency of self-reported long COVID symptoms among participants reporting to suffer from at least one long COVID symptom (*n* = 33).

**Fig. 3:**
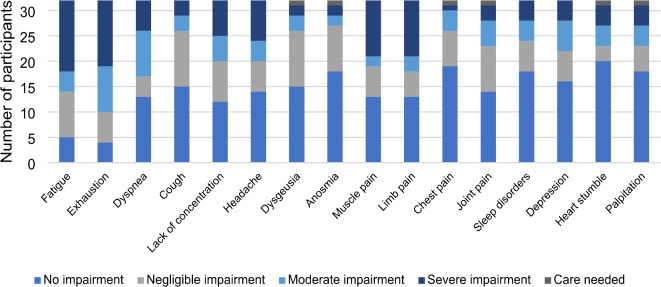
Impact of reported long COVID symptoms on daily activity among participants reporting to suffer from at least one long COVID symptom (*n* = 33).

On average, participants with long COVID reported 5.5 symptoms. The frequencies of each symptom are illustrated in Fig. 2. The most common symptoms were exhaustion, fatigue, and lack of concentration and the most common severe symptoms were fatigue, exhaustion, limb and muscle pain. The impact of the reported long COVID symptoms on daily activity is shown in Table 2; Fig. 3.

**Table 2:** Frequency and severity of symptoms among participants reporting to suffer from at least one long COVID symptom (*n* = 33).

	Impact of reported long COVID symptoms on daily activity				
	No impairment	Negligible impairment	Moderate impairment	Severe impairment	Care needed
Palpitation	18 (56.2%)	5 (15.6%)	4 (12.5%)	4 (12.5%)	1 (3.1%)
Heart stumble	20 (62.5%)	3 (9.4%)	4 (12.5%)	4 (12.5%)	1 (3.1%)
Depression	16 (50.0%)	6 (18.8%)	6 (18.8%)	4 (12.5%)	0 (0.0%)
Sleeping disorder	18 (56.2%)	6 (18.8%)	4 (12.5%)	4 (12.5%)	0 (0.0%)
Joint pain	14 (43.8%)	9 (28.1%)	5 (15.6%)	3 (9.4%)	1 (3.1%)
Chest pain	19 (59.4%)	7 (21.9%)	4 (12.5%)	1 (3.1%)	1 (3.1%)
Limb pain	13 (40.6%)	5 (15.6%)	3 (9.4%)	11 (34.4%)	0 (0.0%)
Muscle pain	13 (40.6%)	6 (18.8%)	2 (6.2%)	11 (34.4%)	0 (0.0%)
Anosmia	18 (56.2%)	9 (28.1%)	2 (6.2%)	2 (6.2%)	1 (3.1%)
Dysgeusia	15 (46.9%)	11 (34.4%)	3 (9.4%)	2 (6.2%)	1 (3.1%)
Headache	14 (43.8%)	6 (18.8%)	4 (12.5%)	8 (25.0%)	0 (0.0%)
Lack of concentration	12 (37.5%)	8 (25.0%)	5 (15.6%)	7 (21.9%)	0 (0.0%)
Cough	15 (46.9%)	11 (34.4%)	3 (9.4%)	3 (9.4%)	0 (0.0%)
Dyspnea	13 (40.6%)	4 (12.5%)	9 (28.1%)	6 (18.8%)	0 (0.0%)
Exhaustion	4 (12.5%)	6 (18.8%)	9 (28.1%)	13 (40.6%)	0 (0.0%)
Fatigue	5 (15.6%)	9 (28.1%)	4 (12.5%)	14 (43.8%)	0 (0.0%)

Data are expressed as absolute numbers and percentages.

we have calculated a severity sum-score, where we summed up all 16 items. For “no impairment” one point was added, for “negligible impairment” two points were added, for “moderate impairment” three points were added, for “severe impairment” four points were added, and for “care needed” five points were added. The resulting severity score had a median of 32.5 points with an interquartile range from 25.5 to 43.5 points in individuals with at least one long COVID symptom. In linear regression models, sex (male vs. female: β = −2.0; 95% confidence interval [CI] = −13.7 to 9.7; *p* = 0.726), vaccination status (no vs. yes: β = 4.2; 95%-CI = −5.8 to 14.2; *p* = 0.396), and occupational group (dental assistant or dental hygienists vs. dentist: β = 3.4; 95%-CI = −4.9 to 11.6; *p* = 0.409) were not significantly associated with COVID symptoms in the long COVID group.

## Discussion

Vaccination rates among the dental team members in Germany were much higher than in the general population. The vaccination rate in the dental team was very high, 82% of the team had at least 3 vaccinations. By contrast, by April 2023 62.6% of the German population had been vaccinated twice and only 15.2% had been vaccinated three times^25^.

The dental team witnessed a turning point in March-April 2020 after the outbreak of the pandemic. Forced closure and shortage of personal protective equipment caused fear and anxiety among dental teams and their patients^26^. Authorization of the first vaccine promised a milder course of disease and enhanced protection from future infection^27^. This achievement was embraced with optimism, offering a relief in time of uncertainty. Despite emerging reports of serious side effects following vaccine administration, dentists and their teams were not reluctant to follow the recommendations of national and international health agencies^28^. The acceptance rate of the COVID vaccine varied greatly across countries (33-97.5%), showing a significant positive association between country income and vaccine acceptance^29,30^. Other studies suggest that the intention to be vaccinated is correlated with the perceived risk of infection^31^. Globally, the perceived risk of infection among dental healthcare personnel was extremely high^32,33^, which explains the notably high percentage of vaccination rate among our sample compared to the general population.

In our sample, we noticed a slightly higher vaccination rate among dentists (95.5%) compared to 84.2% among dental auxiliary personnel. Vaccine hesitancy among healthcare workers is a known complicated multidimensional matter. In Europe, previous research has shown that low socioeconomic status has been a significant obstacle to vaccination against various diseases^34,35^. Additionally, an access to scientifically accurate and up-to-date vaccine information leads to a positive attitude towards vaccination^36^. Our results align with various studies that reported higher vaccination rates among general practitioners and dentists compared to nurses and dental assistants^37^. Although there is not a definite explanation for this phenomenon, several factors may contribute to the differences between these two crucial occupational groups. We presume that a lower socioeconomic status, less vaccination literacy, adverse influence by media coverage, and less trust in pharmaceutical industry / health authorities might be possible explanations for the lower vaccination rates among dental auxiliary personnel.

Unfortunately, there are multiple definitions for long COVID, varying in their time frame for symptoms onset and duration. The WHO defines long COVID symptoms lasting for at least 2 months^11^, whereas the U.S. Centers for Disease Control and Prevention (CDC) requires symptoms to be present for at least 3 months^38^. However, neither organization specifies the nature or number of symptoms required to make a diagnosis. Based on extensive follow-up studies conducted since the outbreak of the pandemic, researchers have compiled a list of over 200 symptoms observed in patients suffering from long COVID^39^. The broad definition of long COVID on one hand and the lack of a specific biomarker or exclusive symptoms on the other hand, poses a challenge for establishing a consensus in research approaches. This variability makes the comparability and generalization of results across different studies particularly difficult. Symptoms of long COVID may resolve over a period of months for most patients or can persist for years^40^. A limitation to our results is that we do not know if our participants have a confirmed medical diagnosis for long COVID or if they fit the global definition. However, our results align with published research examining common long COVID symptoms. Fatigue and exhaustion are the most frequently reported symptoms in our sample (Table 2), leading in almost 40% of the cases to a decrease in daily activities. Interestingly, those symptoms were more pronounced among dental assistants than among dentists. Furthermore, 7 out of 32 (21.9%) participants reported reducing working hours due to long COVID. Several studies have discussed the association between employment status and sickness presenteeism (going to work while being ill)^41^. In the pandemic era, the number of sick notes increased and sick notes were more frequently issued to people with a COVID-19 infection than those without, even in times when most people were already vaccinated^42^. Considering those findings, we can only assume that auxiliary team members tend to take longer to return to work whilst the owner of the practice might return earlier to work.

Reported incidence of long COVID varies greatly (8–35%)^14,39^. Those variation can be attributed to several factors including definition of long COVID, study design, diagnostic methods, targeted demographics and duration of follow-up. Higher incidences were reported among hospitalized cases and particularly among adults aged ≥ 65 years^43^. The exact pathophysiology causing long COVID is still unknown challenging clinicians and researchers alike. Several hypotheses have been presented, each suggesting potential overlapping mechanisms contributing to the persistence of symptoms beyond the acute phase of infection. One hypothesis suggests the presence of a lingering reservoir of SARS-CoV-2 in specific tissues, evading clearance by the immune system and perpetuating ongoing symptoms^44^. Additionally, immunological dysfunction has been implicated as a potential cause of long COVID symptoms^45^. Dysregulated immune responses may result in prolonged inflammation and tissue damage, contributing to the chronicity of symptoms experienced by affected individuals. Furthermore, emerging evidence suggests a potential link between alterations in the gut microbiota and persistence of COVID-19 symptoms^46^. Moreover, the reactivation of underlying pathogens has been proposed as another potential mechanism contributing to the prolonged course of illness observed in some individuals^47^.

We observed a 10% difference in the incidence of long COVID between vaccinated and non-vaccinated dental team members. However, due to the small number of subjects with long COVID, we can not conclusively determine if unvaccinated dental healthcare personnel are more susceptible to suffer from long COVID or experience more severe symptoms. Al-Aly et al.^48^ conducted a comprehensive analysis using data from the electronic healthcare database of the U.S. Department of Veterans Affairs examining long COVID symptoms 6-months post infection in 33,940 individuals with a breakthrough COVID infection and several controls without evidence of COVID infection, including contemporary (*n* = 4,983,491), historical (*n* = 5,785,273) and vaccinated (*n* = 2,566,369) controls. The study compared the risk for long COVID symptoms among vaccinated and unvaccinated participants. Participants who were previously vaccinated showed lower risk of developing 24 of the 47 examined symptoms. This risk mitigation became more evident as the care setting changed from non-hospitalized to requiring ICU-admission. Our results confirm established knowledge and are in line with the findings from Al-Aly et al.^48^. However, it is crucial to acknowledge that while vaccination offers partial protection against the development of long COVID and mortality, it does not guarantee complete immunity. Consequently, relying solely on vaccination to contain the consequences of COVID infection would not be an optimal approach.

Our study focused on a specific occupational cohort. To our knowledge, this is the first study to examine the prevalence and severity of long COVID among dental healthcare personnel in Germany. As a follow-up investigation, this study has some limitations. The selection of our participants was not representative, because we were bound by the informed consent of participants of our baseline examination. Another larger limitation is the low response rate for the follow-up questionnaire. Furthermore, data on symptoms of long COVID were based on self-reported response and may not be medically confirmed. It was also not possible to accurately calculate the time from infection to onset of symptoms of long COVID and how long it lasted. Despite these limitations, our study offers valuable insights into the prevalence and characteristics of long COVID within the dental healthcare sector in Germany. For following projects, it would be interesting to focus on individuals reporting long COVID symptoms who have received professional medical therapy and to examine its long-term effects.

### Conclusion and clinical implication

Our findings indicate that vaccination rates were lower among dental auxiliary personnel compared to dentists. Furthermore, individuals experiencing long COVID symptoms were more frequently dental assistants or dental hygienists than dentists when compared to those without long COVID symptoms. Additionally, our results suggest that dental healthcare personnel are not at a higher risk of experiencing more severe long COVID symptoms than the general population.

## Electronic supplementary material

Below is the link to the electronic supplementary material.

Supplementary Material 1

## Data Availability

The datasets generated during and/or analyzed during the current study are not publicly available due to the data protection act. Deidentified individual participant data that underlie the results reported in this manuscript will be available to the scientific community on request. Applicants interested must provide a proposal form which entails the scientific aim of the usage of the data provided and the institutional review board approval of the research proposal. All proposals should be directed to the corresponding author.
