# Supplementary figures and images for: Vaccination rate and symptoms of long COVID among dental teams in Germany
Source: Sci Rep. 2025 Apr 21;15:13654. doi: 10.1038/s41598-025-96670-8 (PMC12009985; doi:10.1038/s41598-025-96670-8)

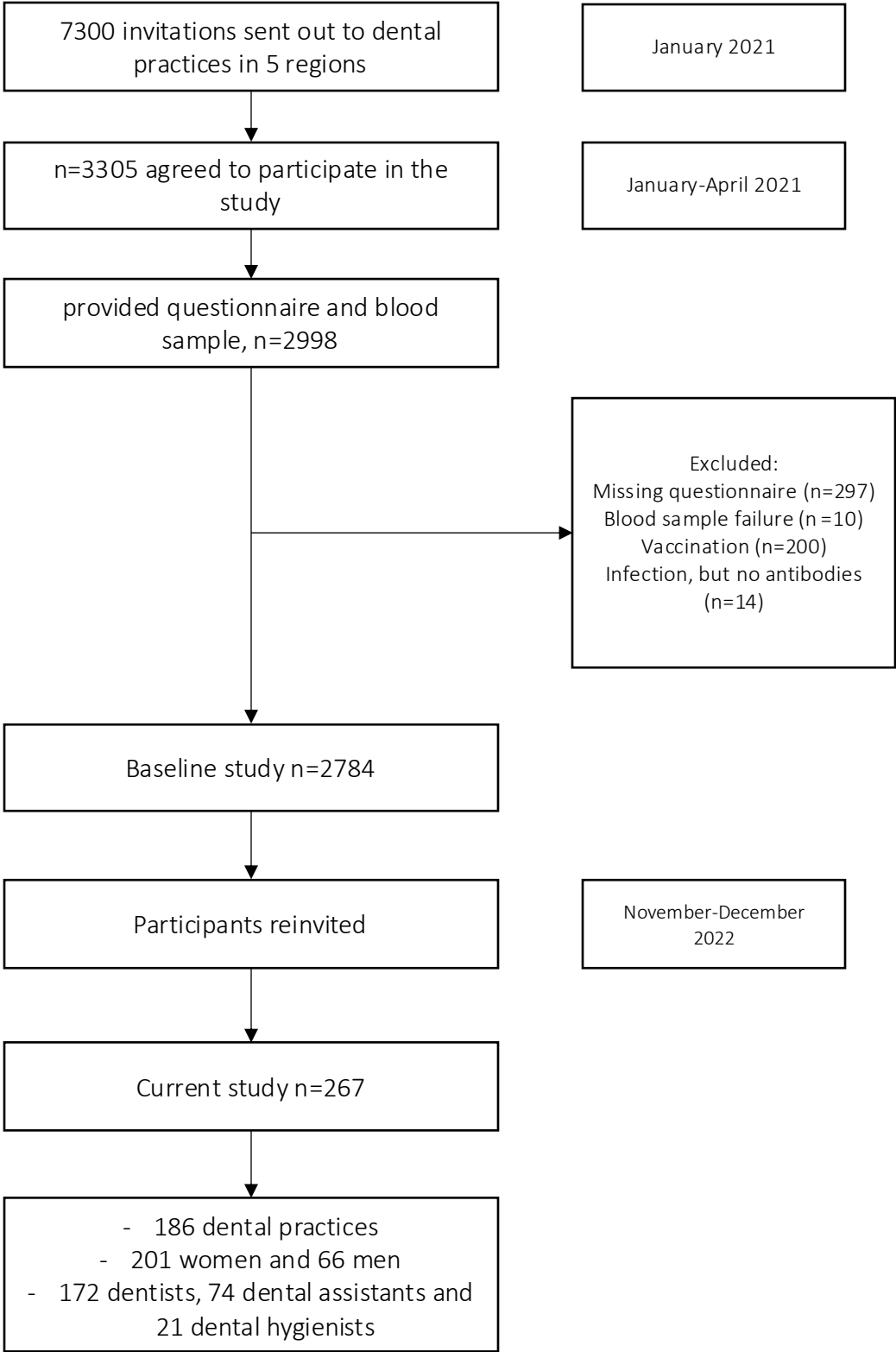

Supplement: Supplementary file 1 — Supplementary Material 1 [file 41598_2025_96670_MOESM1_ESM.pdf]
